# Genomic analysis reveals two dominant strains of *Ornithobacterium rhinotracheale* in Austria and Hungary with distinct multidrug resistance profiles

**DOI:** 10.1128/aem.00569-25

**Published:** 2025-07-21

**Authors:** Nicola Palmieri, Claudia Hess, Boglárka Pollák, Tibor Magyar, Krisztina Pinter, Marianna Doman, Ivana Bilic, Michael Hess

**Affiliations:** 1 Clinic for Poultry and Fish Medicine, Department for Farm Animals and Veterinary Public Health, University of Veterinary Medicine 572162, Vienna, Austria; 2 LVA GmbH 273913 , Klosterneuburg, Austria; 3 National Centre for Public Health and Pharmacy (NNGYK) 72057 , Budapest, Hungary; 4 HUN-REN Veterinary Medical Research Institute 117039 , Budapest, Hungary; 5 National Laboratory of Infectious Animal Diseases, Antimicrobial Resistance, Veterinary Public Health and Food Chain Safety, University of Veterinary Medicine 72408, Budapest, Hungary; Centers for Disease Control and Prevention, Atlanta, Georgia, USA

**Keywords:** *Ornithobacterium rhinotracheale*, multidrug resistance, whole-genome sequencing, phylogenetics, resistance genes, mobile genetic elements, GWAS

## Abstract

*Ornithobacterium rhinotracheale* (ORT) is an emerging pathogen that poses significant challenges to commercial poultry production due to respiratory and systemic infections, leading to economic losses. Increasing multidrug resistance further complicates effective treatment strategies. Here, we present the first large-scale genomic analysis of ORT isolates from Europe, focusing on 94 newly sequenced isolates from Austria and Hungary. Phylogenetic analysis revealed two dominant clades, O1 and O2, which included isolates from both regions, indicating a lack of geographic segregation. Both clades display high genetic similarity, indicating that each represents a single strain. Notably, Clade O1 appears to be specific to Europe and exhibits higher resistance to several antibiotics compared to Clade O2. Genome-wide association studies and database screening identified resistance genes linked to beta-lactam antibiotics (*ccrA*/*orr*) and tylosin (*ermF*/*ermD*). Despite the widespread presence of *tetX* and *tetQ*, these genes did not consistently predict tetracycline resistance. Furthermore, we identified the insertion sequences IS*4351* and IS*1380* as key mobile elements mediating the co-localization of *tetX* and *ermF*/*ermD* resistance genes within ORT isolates of Clade O1. These findings enhance our understanding of ORT’s genetic diversity, strain distribution, and resistance mechanisms, providing a basis for improved monitoring, vaccine development, and targeted interventions. The limited genetic variability observed in our data set underscores the challenges of identifying resistance genes and highlights the need for novel approaches to fully elucidate resistance mechanisms.

**IMPORTANCE:**

*Ornithobacterium rhinotracheale* (ORT) is a bacterial pathogen that causes significant respiratory and systemic disease in poultry, leading to economic losses worldwide. By sequencing and comparing 94 isolates from Austria and Hungary, we discovered two major ORT strains circulating in these regions. One strain is unique to Europe and carries multiple antibiotic resistance genes on a mobile element, underscoring the need for careful antibiotic use. This study is the first large-scale genomic analysis of European ORT isolates, revealing how these strains spread and evolve over time. Our findings offer valuable insights for improving diagnostic methods, guiding vaccine development, and designing targeted strategies to manage ORT infections in commercial poultry flocks.

## INTRODUCTION

*Ornithobacterium rhinotracheale* (ORT) has emerged as a significant pathogen affecting commercial avian species, leading to respiratory and systemic infections (1). These infections pose a substantial economic burden on poultry health worldwide. Impact on production is due to reduced food intake, weight loss, reduced egg production, and increased mortality rates (2). Transmission is primarily through inhalation and direct contact (3), but field data also suggest possible vertical transmission (3, 4).

One area of particular concern is the inconsistency and failure of antibiotic treatments for ORT, often attributed to the development of multidrug resistance (5–7). Previous research has focused on the patterns of antibiotic resistance in ORT isolates but has not investigated the genetic basis of this resistance (8–14). Early studies, such as the one by Devriese et al. (8), highlighted the complexities in establishing antibiotic sensitivity due to the organism’s intricate growth requirements. Similarly, Soriano et al. (9) found a marked trend toward antimicrobial drug resistance among Mexican isolates. Studies conducted on isolates obtained from Minnesota in 1996–2002 revealed annual increasing resistance trends against various antibiotics, emphasizing the need for continued monitoring (10). In Hungary, Szabó et al. (11) found that most ORT strains were resistant to nalidixic acid, sulfamethoxazole-trimethoprim, and gentamicin. A similar trend of multidrug resistance was observed in studies conducted in Mexico (12) and Iran (13, 14), with isolates showing resistance to ampicillin, erythromycin, ceftriaxone, and penicillin. The recent study by Hess et al. (5) added to the growing concern by demonstrating the enduring nature of antibiotic resistance in Austrian ORT isolates over a 20 year period. Such isolates were predominantly resistant to aminoglycosides and colistin while showing susceptibility to penicillins and cephalosporins. Importantly, all isolates were classified as multidrug-resistant, reaffirming the urgency of this issue.

The persistence of multidrug resistance among ORT isolates emphasizes the need to explore the genetic mechanisms driving this resistance. Genomic studies offer a powerful tool for uncovering these mechanisms, providing insights into the genetic basis of antibiotic resistance and the broader evolutionary dynamics of ORT. Early genome sequencing studies have provided foundational knowledge about ORT. For example, the genomes of ORT strains H06-030791 and ORT-UMN 88, both consisting of circular chromosomes of over 2.3 million base pairs, have been sequenced and annotated (15, 16). These genomes have helped to identify key protein-coding genes and RNA genes, laying the groundwork for understanding the bacterium’s core and pan-genomes. One pivotal study has made a significant contribution to our understanding of the comparative genomics and evolutionary dynamics of ORT. Smith and colleagues (17) conducted an extensive analysis involving whole-genome sequencing of 157 clinical isolates originating from turkey flocks from various producers in the USA to identify single nucleotide polymorphisms (SNPs) and putative virulence factors. This approach has highlighted the existence of multiple ORT strains circulating within the same turkey operation, thus underlining the complexity of ORT’s evolutionary dynamics. Advanced sequencing methods, such as Nanopore-Illumina hybrid assembly, have further expanded our genomic understanding of ORT (18). Additionally, new strains that deviate significantly from known reference strains have been identified, challenging our current taxonomic understanding of ORT (19).

While some progress has been made in understanding the genomic landscape of ORT, significant knowledge gaps remain, particularly concerning the genetic diversity and spread of ORT strains in Europe. ORT is known to be a prominent pathogen in Europe (14, 15), yet genomic studies focusing on European isolates are limited (19). To address this gap, we have sequenced and analyzed 94 ORT isolates from Austria and Hungary, providing the first large-scale genomic study of European ORT isolates. Our data set enabled us to monitor genetic diversity, assess intra- and inter-farm variation, and identify potential resistance mechanisms in these populations.

## MATERIALS AND METHODS

### Data set composition and metadata

A total of 45 Austrian isolates were derived from a previous study by Hess et al. (5), which investigated antimicrobial susceptibility profiles and multidrug resistance in ORT isolated from turkeys over a 20 year period. The isolate names for the Austrian samples follow the format [year]_[isolate_id]. Additionally, we incorporated 49 newly sequenced isolates from Hungary, including 55 collected from 2001 to 2023 and 4 older isolates from 1991 to 1995, to enrich our data set. The isolate names for Hungarian samples consist of four-digit numeric identifiers that do not represent collection years. This resulted in a total of 94 isolates, including both Austrian and Hungarian samples. Antimicrobial susceptibility testing was performed according to Hess et al. (5). For this, a commercially available microdilution assay was used (MERLIN Diagnostika GmbH, Bornheim-Hersel, Germany) which included 19 antimicrobial substances belonging to 10 classes. Comprehensive metadata were curated for each isolate, encompassing details such as collection year, isolation country, host, genome metrics for the Illumina-based assemblies, antibiotic resistance profiles, and presence/absence patterns for resistance genes (Table S1).

### Bacterial culture and DNA extraction

Austrian isolates were grown on blood agar (COS, BioMérieux) under microaerophilic conditions for 24–48 h. Afterward, culture material was collected and suspended in 2 mL of phosphate-buffered saline (GIBCO). The suspension was centrifuged, and the pellet was used for DNA extraction. For Illumina sequencing, DNA was extracted using the DNeasy Blood and Tissue Kit (Qiagen, Hilden, Germany) following the manufacturer’s instructions. For Oxford Nanopore Technology sequencing, high molecular weight DNA was extracted using a phenol/chloroform method as previously described (19). Hungarian isolates were cultured in Tryptic Soy Broth supplemented with 5% yeast extract and 5% fetal bovine serum and incubated at 37°C for 48 h under microaerophilic conditions. Genomic DNA extraction was carried out using the Quick-DNA Fungal/Bacterial Miniprep Kit (Zymo Research, USA) following the manufacturer’s instructions.

### Genome sequencing

Sequencing for Austrian isolates was performed on the Illumina MiSeq platform (VBCF NGS Facility, Vienna, Austria), producing 150 bp paired-end reads. Additionally, three Austrian isolates (2003_03567, 2009_16066, and 2013_01156) were resequenced using Nanopore MinION to obtain long reads. Nanopore sequencing libraries were prepared from 1 µg of high molecular weight genomic DNA using the Native Barcoding Kit V14 (SQK-NBD114.24, Oxford Nanopore Technology, Oxford, UK), allowing multiplexing of all three isolates on a single MinION R10.4 flow cell, generating kb-sized long reads. Hungarian isolates were also sequenced on the Illumina MiSeq platform, generating 150 bp paired-end reads. Sequencing was performed by SeqOmics Biotechnology Ltd. (Mórahalom, Hungary).

### Genome assembly

Raw sequence reads from Illumina sequencing were quality checked and trimmed using Cutadapt (20) with parameters -a AGATCGGAAGAGC -A AGATCGGAAGAGC -m 20 0 to remove any adapter sequences and low-quality reads. The trimmed reads were then assembled into genomes using the *De Novo* Assembly tool from CLC Genomics Workbench v.23.0.4 with default parameters. After assembly, ORT-specific contigs were extracted using Kraken2 (21) against a custom ORT database derived from all the high-quality ORT genomes from NCBI, which included the following 14 isolates: 46, 97, A171, C183, DSM_15997, FARPER-174b, H06-030791, IRI-Sh, K223, LMG_18856, LMG_18861, LMG_19032, OR14, and ORT-UMN_88. Kraken2 was run with the -–classified-out parameter to retain only contigs classified as *O. rhinotracheale*. To tailor gene prediction to the target organism, we first generated a custom training file using Prodigal (22) with the DSM_15997 reference strain genome as input, applying the parameters -c -m -g 11 p single -q. This custom training file was then used in PROKKA (23) for genome annotation, with the parameters --compliant --genus Ornithobacterium --species rhinotracheale --prodigaltf prodigal_training_file. Plasmid sequences were annotated using MOB-suite v.3.1.9 (24) with default parameters.

### Phylogenetic analysis

To investigate the genetic relationships and clustering patterns among our ORT isolates, we constructed a phylogenetic tree using Parsnp v.1.2 (25), based on ORT-classified contigs from the Illumina assemblies. The reference genome DSM_15997 was used for alignment, and the analysis included the -x parameter to exclude SNPs from recombinant regions. Additionally, pairwise average nucleotide identity (ANI) was computed using FastANI (26) to quantify genetic similarity among the Austrian and Hungarian ORT isolates. To compare our ORT isolates with US isolates from Smith et al. (17), sequencing reads from the BioProject PRJNA524749 were retrieved and assembled them into contigs using the De Novo Assembly module in CLC Genomics Workbench v.24. Genome quality was assessed with CheckM (27), and assemblies with contamination levels exceeding 5% were excluded from further analysis. FastANI (26) was used to compute pairwise ANI across the assembled genomes.

### Identification of antibiotic resistance genes

#### Identification by database screening

For a comprehensive identification of antibiotic resistance through database screening, all isolate gene sequences annotated by PROKKA were screened against three major antimicrobial resistance databases: MEGARes v.3.0 (28), CARD (29), and AMRFinder (30). For both MEGARes and CARD, we performed BLASTN searches using all predicted genes from each isolate, applying a minimum sequence identity of 90% and a minimum query coverage of 60%. For AMRFinder, we ran the tool on each isolate’s gene sequence, specifying the Prokka annotation format and applying analogous coverage and identity thresholds (-c 0.6, -i 0.9). We adopted these thresholds to align with the parameters used in ResFinder (http://genepi.food.dtu.dk/resfinder), a widely used program to find resistance genes, ensuring consistency and reliability in the identification of resistance markers. To enable direct comparison across methods, results from the three tools were merged based on the unique gene identifiers assigned by PROKKA.

#### Identification by genome-wide association study (GWAS)

To identify genes associated with antibiotic resistance through GWAS, we generated the phylogenetic tree using IQTREE (31) and ClonalFrameML (32) as recommended by the authors of treeWAS (33) to increase accuracy. We encoded the phenotype as binary, where resistant (R) and intermediate (I) were assigned a value of 1, and sensitive (S) was assigned a value of 0. We conducted three types of GWAS: (1) gene-based GWAS using treeWAS: We used the phylogenetic tree generated and gene presence-absence data derived from Panaroo (34) with parameters --mode strict --remove_by_consensus True. The treeWAS parameters were set with p_value = 0.05 and p_value_correct = “bonf.” The GWAS output was filtered by considering only genes with a prevalence greater than 50% in resistant isolates and less than 50% in sensitive isolates; (ii); gene-based GWAS using a phi coefficient: we computed the phi coefficient between each binary vector representing antibiotic resistance (R/I = 1, S = 0) and each gene’s binary profile (0 for absence, 1 for presence), selecting genes with a phi coefficient greater than 0.7; (iii) SNP-based GWAS using treeWAS: we performed SNP-based GWAS with treeWAS using the phylogenetic tree generated and SNPs calculated from the Parsnp tree used for phylogenetic reconstruction. The treeWAS parameters were set with p_value = 0.05 and p_value_correct = “bonf.” Multiallelic SNPs were recoded using a multi-line representation (35). The resulting SNPs from treeWAS were annotated using SnpEff. Furthermore, we filtered the SNPs by considering only those with a prevalence greater than 50% in resistant isolates and less than 50% in sensitive isolates. We did not perform SNP-based GWAS using the phi coefficient as it led to a substantial number of false positives, attributed to the strong linkage between resistance profiles and phylogenetic clades observed in this data set.

#### Analysis of genomic context and co-localization of *tetX* and *ermF*

To analyze the genomic context of *tetX* and *ermF*, isolates containing both genes were identified based on PROKKA annotations. For each of these isolates, contigs containing *tetX* and *ermF* were visually inspected to assess contig length and gene positioning relative to contig borders. Genomes were aligned using the “Create Whole Genome Alignment” tool from the CLC Genomics Workbench plugin Whole Genome Alignment. Putative transposase flanking sequences were annotated based on PROKKA output and further queried using BLASTN against the NCBI nt database (restricted to bacterial sequences; ≥90% identity and ≥ 90% query coverage) to determine their identity. To investigate the genomic origin of the *tetX–ermF* region, three isolates were resequenced using Nanopore MinION. Raw Nanopore reads from those isolates were basecalled using MinKNOW version 24.11.10 (Oxford Nanopore Technologies) and assembled using Flye v.2.9 (36) with the --nano-hq option and default parameters. Contigs containing *tetX* and *ermF* from these Nanopore assemblies, along with contigs from Illumina assemblies where both genes were found on long contigs and not located at contig borders, were manually extracted using CLC Genomics Workbench. The extracted regions were aligned and visualized using clinker (37) to compare gene content and synteny. Representative *tetX–ermF* regions containing flanking insertion sequences were also queried against the NCBI nt database using BLASTN with ≥90% identity and ≥90% query coverage.

## RESULTS

### Genome assembly

**Fig 1:**
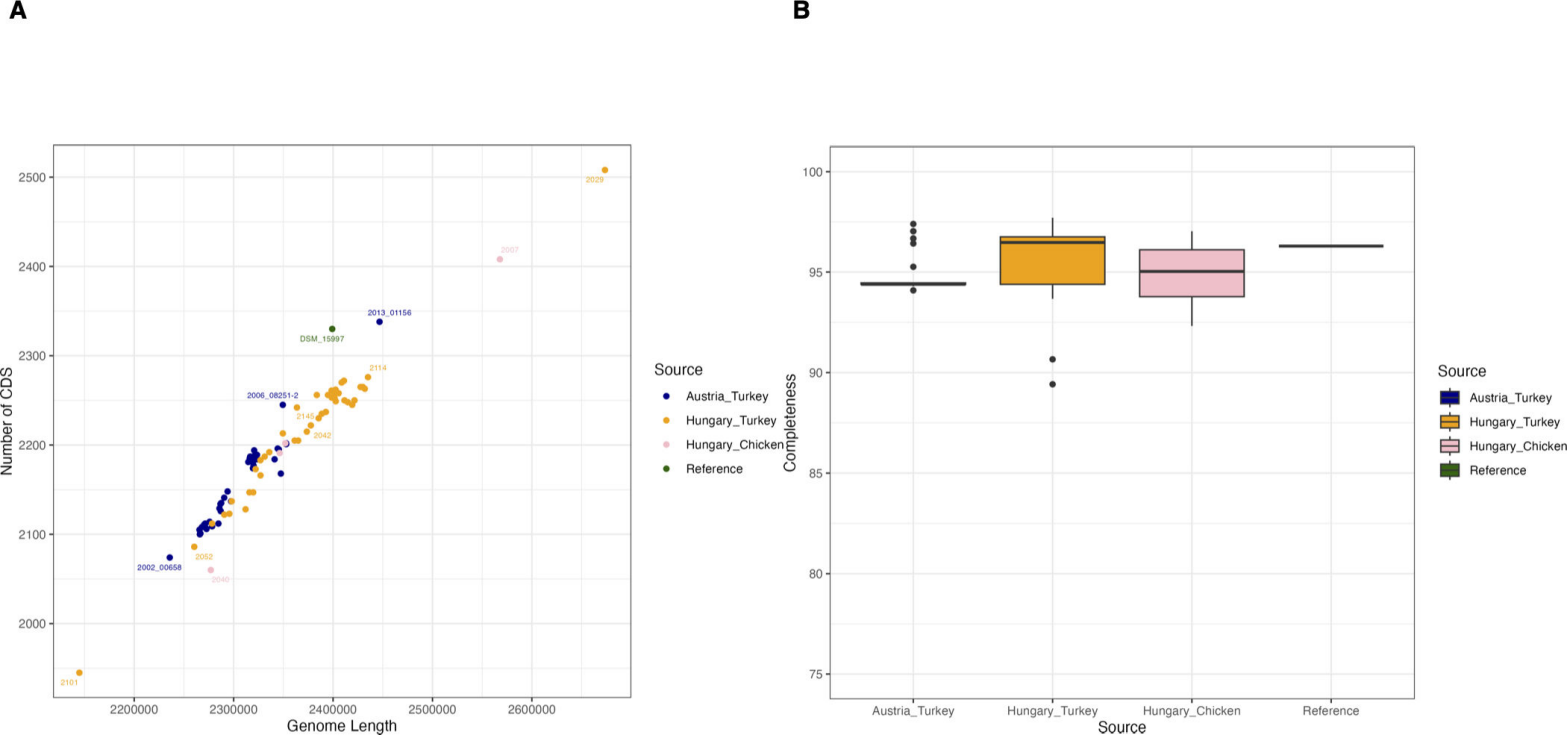
Comparative analysis of ORT genome assemblies. (**A**) Relationship between genome length and the number of coding sequences (CDS) for 94 ORT isolates and the reference genome DSM_15997. (**B**) Boxplots of genome completeness values across different groups of isolates.

To better understand ORT and elucidate diversity within and between farms, as well as to identify antibiotic resistance genes, we sequenced 45 Austrian isolates from turkeys collected between 2002 and 2020 as described by Hess et al. (5). This data set was further expanded with 49 additional Hungarian isolates, comprising 45 turkey isolates and four chicken isolates collected between 1991 and 2023, along with the reference genome DSM_15997. In total, our data set consisted of 94 newly sequenced isolates plus the reference genome. All genomes were assembled from Illumina sequencing data. We observed that the Austrian isolates tended to have shorter genome lengths compared to the Hungarian isolates (Fig. 1A). This is attributed to their lower sequencing coverage. However, CheckM analysis showed that all isolates, including the Austrian ones, had completeness values well above 90% (Fig. 1B), indicating that all genomes are suitable for downstream analyses. Plasmid detection analysis with MOB-suite (24) did not identify plasmid sequences in any of the assemblies.

### Phylogenetic analysis

#### Phylogenetic diversity in Austrian and Hungarian isolates

**Fig 2:**
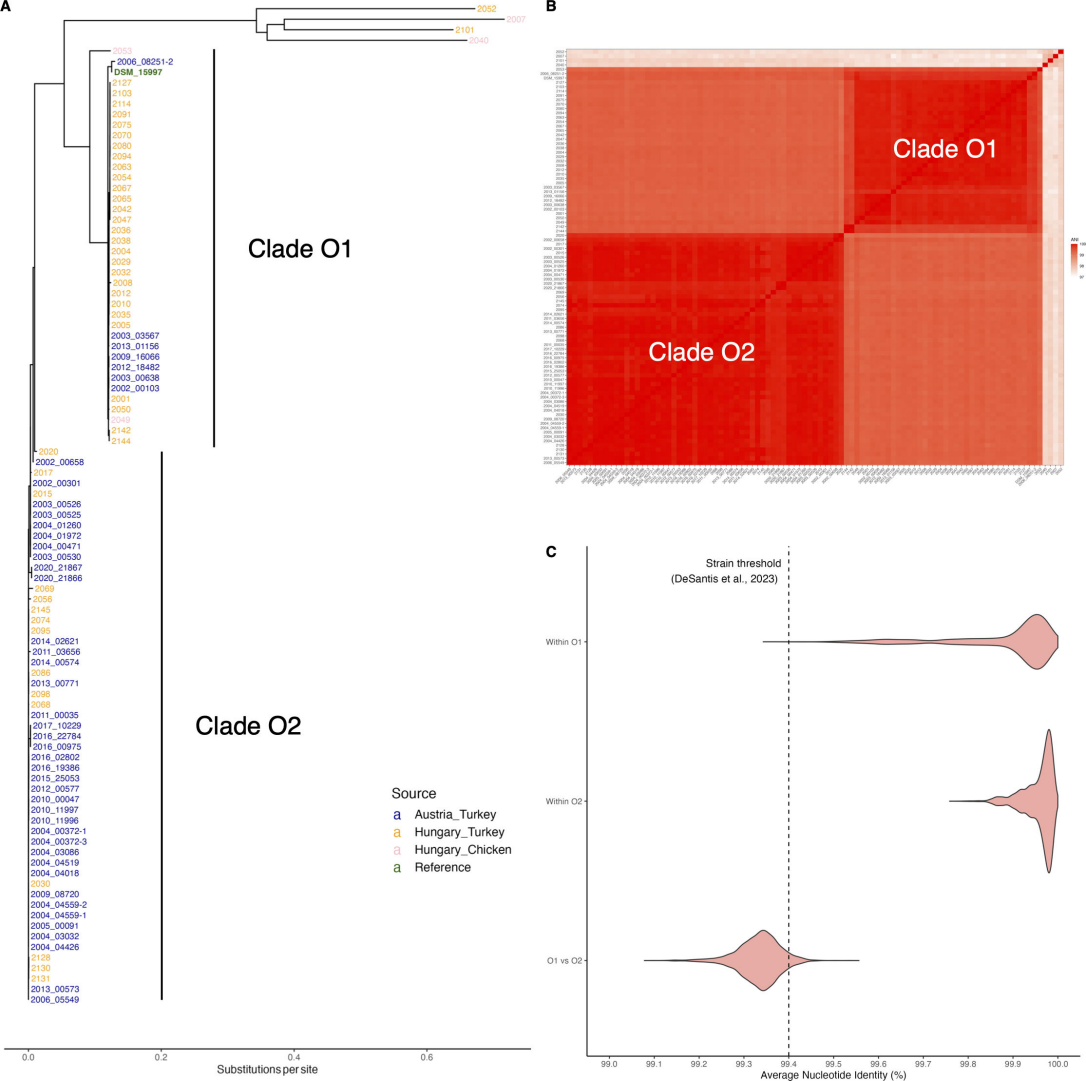
Phylogenetic relationships and genomic similarity among Austrian and Hungarian ORT isolates. (**A**) Phylogenetic tree of 94 newly sequenced ORT isolates from Austria and Hungary, along with the reference strain DSM_15997, constructed using Parsnp. The two dominant clades, O1 and O2, are highlighted. (**B**) ANI heatmap showing pairwise genomic similarity among all isolates. The ANI heatmap shows pairwise comparisons of nucleotide identity, with darker red shades indicating higher ANI values. (C) Violin plot showing the distribution of pairwise ANI values within clades O1 and O2, and between clades. The dashed vertical line indicates the 99.4% ANI threshold proposed by DeSantis et al. (38) as the upper limit for strain-level differentiation.

To elucidate the genetic relationships among our ORT isolates, we conducted a phylogenetic analysis including 94 newly sequenced isolates and the reference genome DSM_15997. The resulting phylogenetic tree revealed two primary clades, O1 and O2, both displaying minimal genetic divergence within each clade, suggesting that each might represent a distinct strain (Fig. 2A). These clades included isolates from both Austria and Hungary, indicating the absence of geographic segregation between the strains. Together, Clades O1 and O2 encompass 90 isolates. Beyond these, four isolates (2025*,* 2007*,* 2101*,* and 2040) each form distinct, independent clades with branch length exceeding 0.6 substitutions per site (Fig. 2A), and are therefore referred to as highly divergent isolates throughout the manuscript. Interestingly, the two older Hungarian chicken isolates (2053 and 2049) were positioned closer to the root of the tree compared to the two more recent Hungarian chicken isolates (2007 and 2040). However, the chicken isolates did not form a cohesive clade, indicating that there is no host-specific clustering within the data set. No correlation between phylogenetic clustering and collection year was found, as isolates from 1991 to 2023 were randomly distributed across clades (Fig. S1). To better define strains, we computed the ANI among the isolates (Fig. 2B). The within-clade ANI values for Clade O1 ranged from 99.5% to 100%, and for Clade O2 from 99.8% to 100%, indicating high homogeneity among the isolates within each clade. The ANI values between Clades O1 and O2 ranged from 99.2% to 99.5%, suggesting that the two clades might represent distinct strains. The four highly divergent isolates (2025*,* 2007*,* 2101*,* and 2040) had ANI values ranging from 97.6% to 98.2% within themselves. The ANI values between these isolates and Clade O1 ranged from 97.2% to 97.8%, and the ANI values between these isolates and Clade O2 ranged from 97.3% to 97.8%, indicating greater genetic divergence and suggesting that they represent separate strains. To determine whether Clades O1 and O2 represent distinct strains or a single clonal lineage, we computed all pairwise ANI values among the 90 isolates in these two clades. Comparisons within O1 ranged from 99.4% to 100% (mean: 99.9%), and within O2 from 99.8% to 100% (mean: 100%). In contrast, O1 vs O2 comparisons ranged from 99.1% to 99.5%, with a mean of 99.3%. This shift in distribution is clearly visible in Fig. 2C, where the three comparison types form separate clusters, and most between-clade values fall below the 99.4% threshold. To interpret these values, we adopted the threshold proposed by DeSantis et al. (38), a large-scale genome-based taxonomy study that explicitly defines 99.4% ANI as the boundary between same-strain and different-strain comparisons. This threshold was derived from more than 80,000 bacterial genome assemblies and represents a well-supported genetic discontinuity between technical replicates and distinct biological isolates. In our data, all within-clade comparisons exceed 99.4%, while the majority of O1 vs O2 comparisons fall below, indicating a shift in ANI values that supports their classification as distinct strains under the proposed boundary. To formally test this differentiation, we first assessed the normality of ANI values within each group. Shapiro-Wilk tests indicated significant deviations from normality for all comparison types (within O1: W = 0.76, *P* < 2.2e – 16; O1 vs O2: W = 0.97, *P* < 2.2e – 16), prompting the use of nonparametric alternatives. A Wilcoxon signed-rank test confirmed that ANI values between O1 and O2 isolates were significantly lower than the 99.4% strain threshold (V = 118,252, *P* < 2.2e – 16), supporting their classification as distinct strains. Additionally, a Kruskal-Wallis test revealed a highly significant difference in ANI across the three comparison types (χ² = 6,332.9, df = 2, *P* < 2.2e – 16). *Post hoc* pairwise Wilcoxon tests, adjusted using the Benjamini-Hochberg method, showed that O1 vs O2 comparisons were significantly different from both within O1 and within O2 comparisons (adjusted *P* < 2e – 16). These results demonstrate that Clades O1 and O2 are genetically distinct according to both operational thresholds and statistical analysis, and therefore should be classified as separate strains.

#### Phylogenetic relationships between Austrian-Hungarian and US isolates

**Fig 3:**
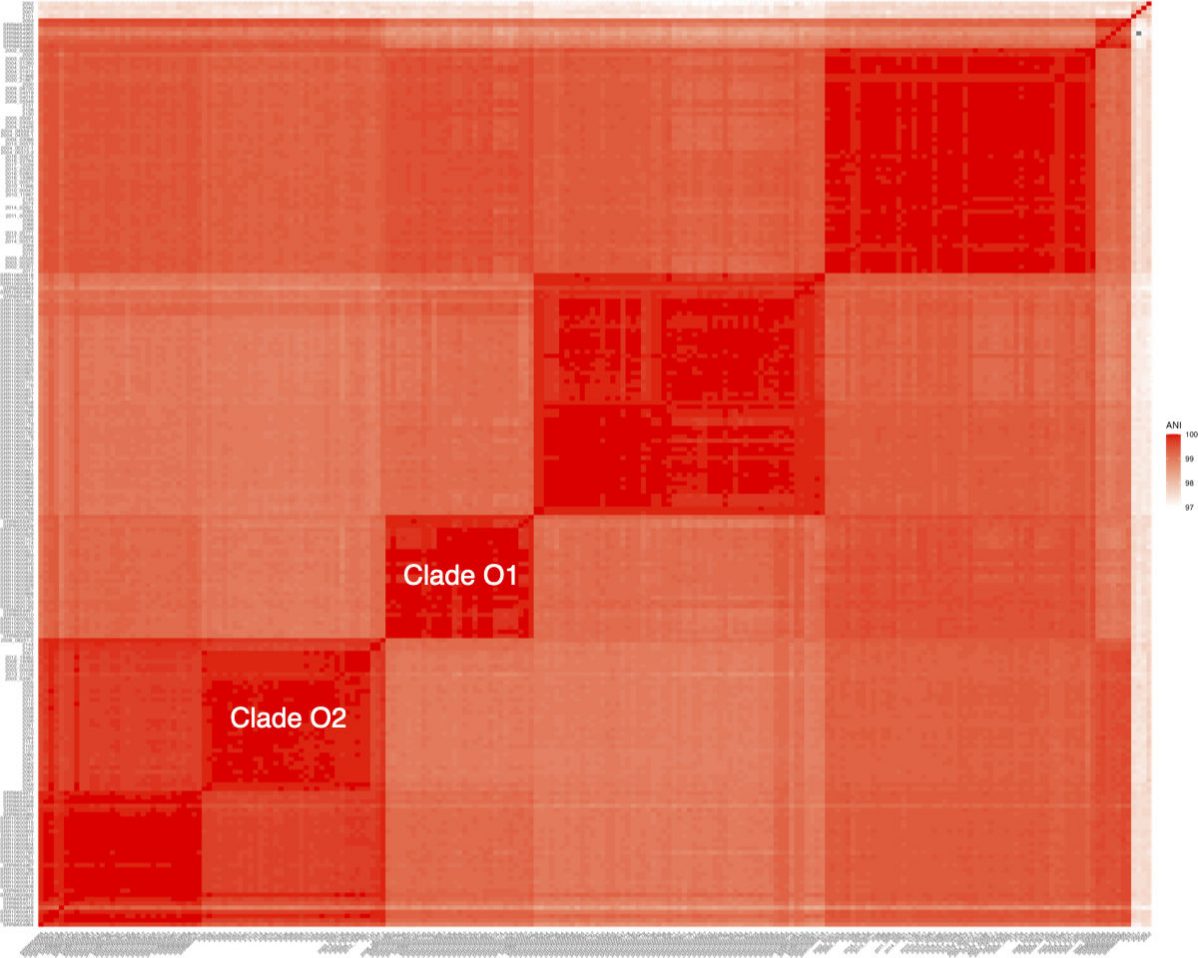
Genomic similarity of Austrian-Hungarian ORT isolates and US isolates from Smith et al. ANI heatmap comparing Austrian-Hungarian and US isolates, illustrating the genetic distinctiveness of Clade O1 and the integration of Clade O2 within a US clade. Darker shades indicate higher ANI values.

A recent large-scale genomic study of ORT in the USA by Smith et al. (17) identified four distinct phylogenetic clades among 157 clinical isolates from commercial turkey farms. To assess how the two dominant Austrian-Hungarian clades (O1 and O2) relate to these US clades, we performed a combined comparative genomic analysis using an ANI heatmap (Fig. 3). This method provides an intuitive visualization, where distinct clusters appear as squares along the diagonal, facilitating the identification of genomic relationships. Since assembled genomes from Smith et al. (17) were not available in the NCBI Genome database at the time of our study, we retrieved the raw sequencing reads and performed *de novo* assembly. During this process, we identified several cases of contamination leading to artificially long assemblies, prompting their removal. As a result, we retained 124 out of the original 157 isolates for analysis. Despite these exclusions, the remaining 124 isolates still encompassed all four US clades defined in their study, allowing for a robust comparative analysis (Fig. 3). Another limitation for this comparison was that strain names from Smith et al. (17) were not available in the tree, preventing a direct mapping of their four clades (Clades 1–4) onto the heatmap. Nonetheless, a clear clustering pattern emerged. Moving from the bottom right along the diagonal (Fig. 3), Clade O2 appears embedded within one of the US clades while also forming a distinguishable subclade. In contrast, Clade O1 remains entirely distinct from the US isolates, forming an independent lineage specific to Europe. The four highly divergent isolates from our data set (2025*,* 2007*,* 2101*,* and 2040) also remain separate from the major clades, clustering distinctly in the top right corner of the heatmap. Notably, Hungarian isolate 2053, visible as the fifth isolate from the top on the *y*-axis of the heatmap, clustered within the smallest US clade, representing the only European isolate that grouped directly with a US clade rather than forming a separate cluster. In summary, the combined data set reveals five primary ORT clades: four corresponding to US clades from Smith et al. (17) and one exclusive to Europe (Clade O1). Additionally, one of the US clades appears to contain a distinct subclade composed of both US and Austrian-Hungarian isolates (Clade O2).

#### Relationship with farms in Austrian isolates

**Fig 4:**
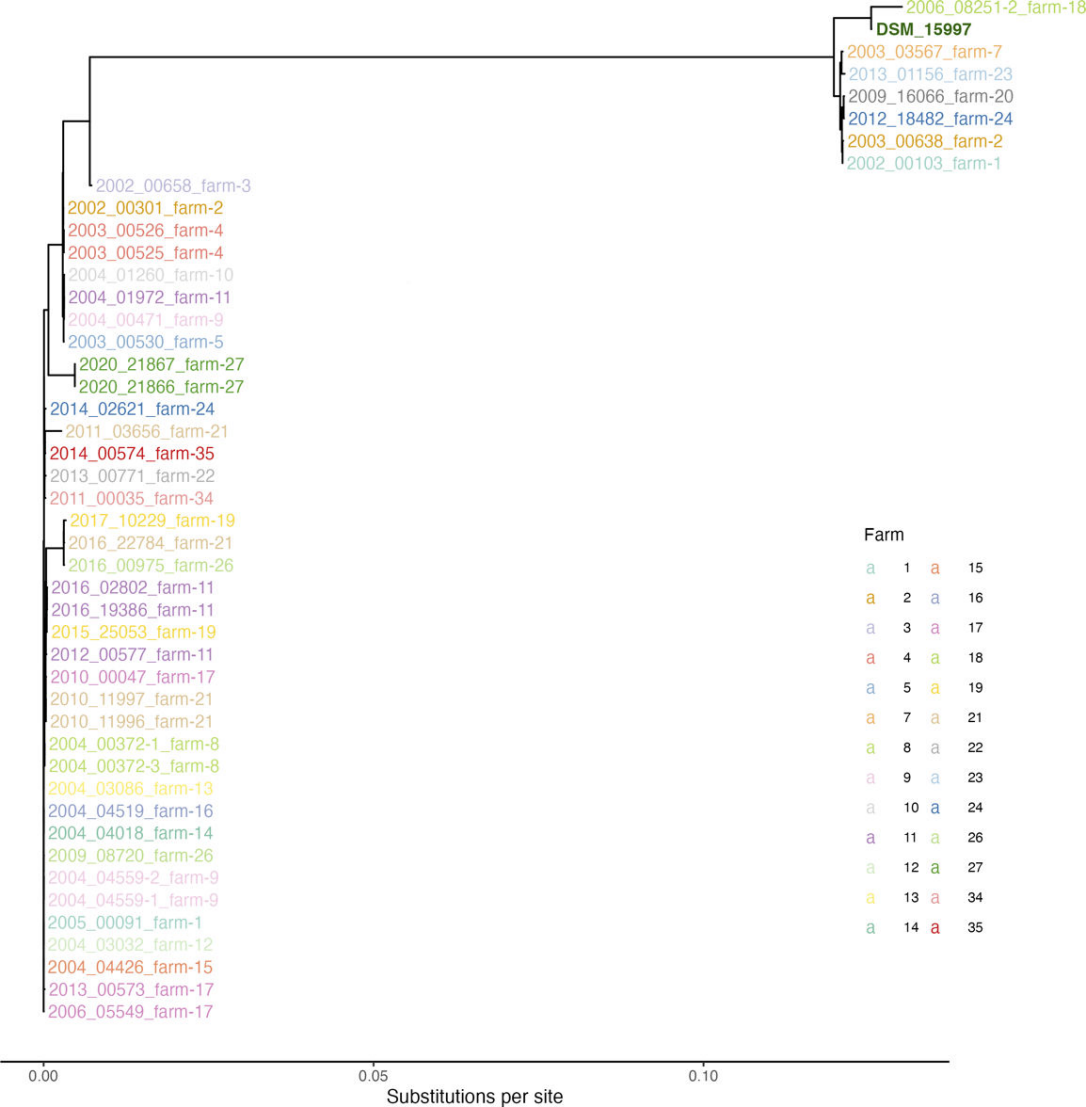
Phylogenetic tree of Austrian ORT isolates with farm-level labeling. Phylogenetic tree of Austrian ORT isolates constructed using Parsnp 1.2, with labels color-coded by farm of origin. The reference genome DSM_15997 is shown in dark green.

We examined the distribution of ORT strains across 27 unique farms in Austria, focusing on the relationship between the phylogenetic clades and farm origin (Fig. 4), since this information was only available for the Austrian isolates. Four farms (7, 18, 20, 23) each had a single isolate belonging to Clade O1, while three farms (24, 2, and 1) contained both Clade O1 and Clade O2 isolates, indicating the presence of intra-farm genetic diversity. The remaining 20 farms exclusively harbored isolates from Clade O2, reflecting a more genetically homogeneous population.

#### Relationship with antibiotic resistance profiles

**Fig 5:**
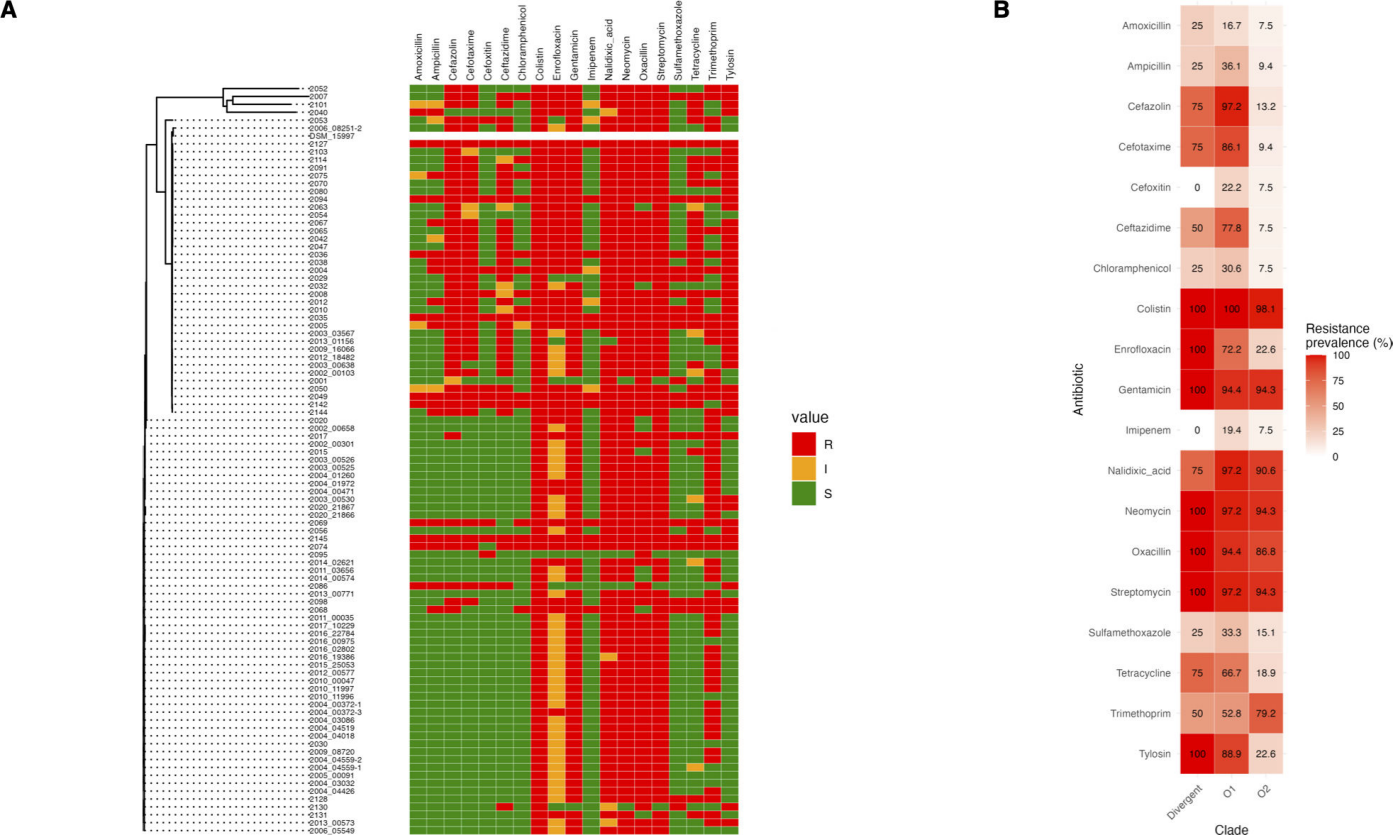
Antibiotic resistance profiles of ORT isolates and their relationship with phylogenetic clades. (**A**) Phylogenetic tree generated with Parsnp 1.2 with corresponding heatmap showing resistance (red), intermediate (yellow), and sensitive (green) phenotypes for 19 antibiotics across all isolates. (**B**) Heatmap showing the percentage of resistant isolates by clade for each antibiotic, with darker shades of red indicating higher percentages of resistance. The “Divergent” category includes the four genetically distinct isolates (2025*,* 2007*,* 2101*,* and 2040) that do not fall within Clade O1 or O2.

We examined the relationship between antibiotic resistance profiles and the phylogenetic clades identified in our data set. This analysis is illustrated in Fig. 5A, which shows the phylogenetic tree alongside the resistance profiles for each isolate. In total, 19 antibiotics were screened for susceptibility. Most isolates were resistant to colistin, gentamicin, nalidixic acid, neomycin, oxacillin, and streptomycin, regardless of phylogenetic clade, indicating widespread resistance to these antibiotics across all isolates. For several antibiotics, including amoxicillin, ampicillin, cefoxitin, chloramphenicol, imipenem, and sulfamethoxazole, the prevalence of resistance was consistently low (below 35%) across all clades, suggesting that most isolates are sensitive to these antibiotics (Fig. 5B). Notably, Clade O1 exhibited a markedly higher prevalence of resistance to cefazolin, cefotaxime, ceftazidime, enrofloxacin, tetracycline, and tylosin compared to Clade O2, indicating distinct resistance patterns between the two main clades. In contrast, Clade O2 showed a higher prevalence of resistance to trimethoprim (79.2%) compared to Clade O1 (52.8%), although resistance to trimethoprim was still relatively common in both clades. The four highly divergent isolates (2025*,* 2007*,* 2101*,* and 2040), labeled as “Divergent” in the heatmap (Fig. 5B), exhibited a resistance profile that was very similar to Clade O1.

### Core genome analysis and known virulence and resistance genes

Across the 94 sequenced isolates from Austria and Hungary, we identified a total of 4,548 genes, of which 1,059 (23.3%) were core genes present in 99% of the genomes. In addition, there were 680 (15.0%) genes found in 95 to 99% of genomes, 760 (16.7%) genes present in 15 to 95% of genomes, and 2,049 (45.0%) classified as accessory genes, being present in fewer than 15% of the genomes. Several virulence factors were encoded in the core genome (17), including the toxin-antitoxin biofilm protein TabA, which plays a role in biofilm formation, and proteins related to gliding motility, such as GldH and MotB, which are thought to facilitate host colonization. Additionally, genes associated with multidrug resistance (17) were highly prevalent across all isolates, including *macA* and *macB* (macrolide resistance), *mdtA*, *mdtC*, *norM*, *norR*, *yheS*, and *yheI*. The *oatA* gene, which has been implicated in beta-lactam resistance (17, 39), was identified only in isolate 2053.

### Detailed analysis of antibiotic resistance genes

#### Identification of resistance genes by database screening

**Fig 6:**
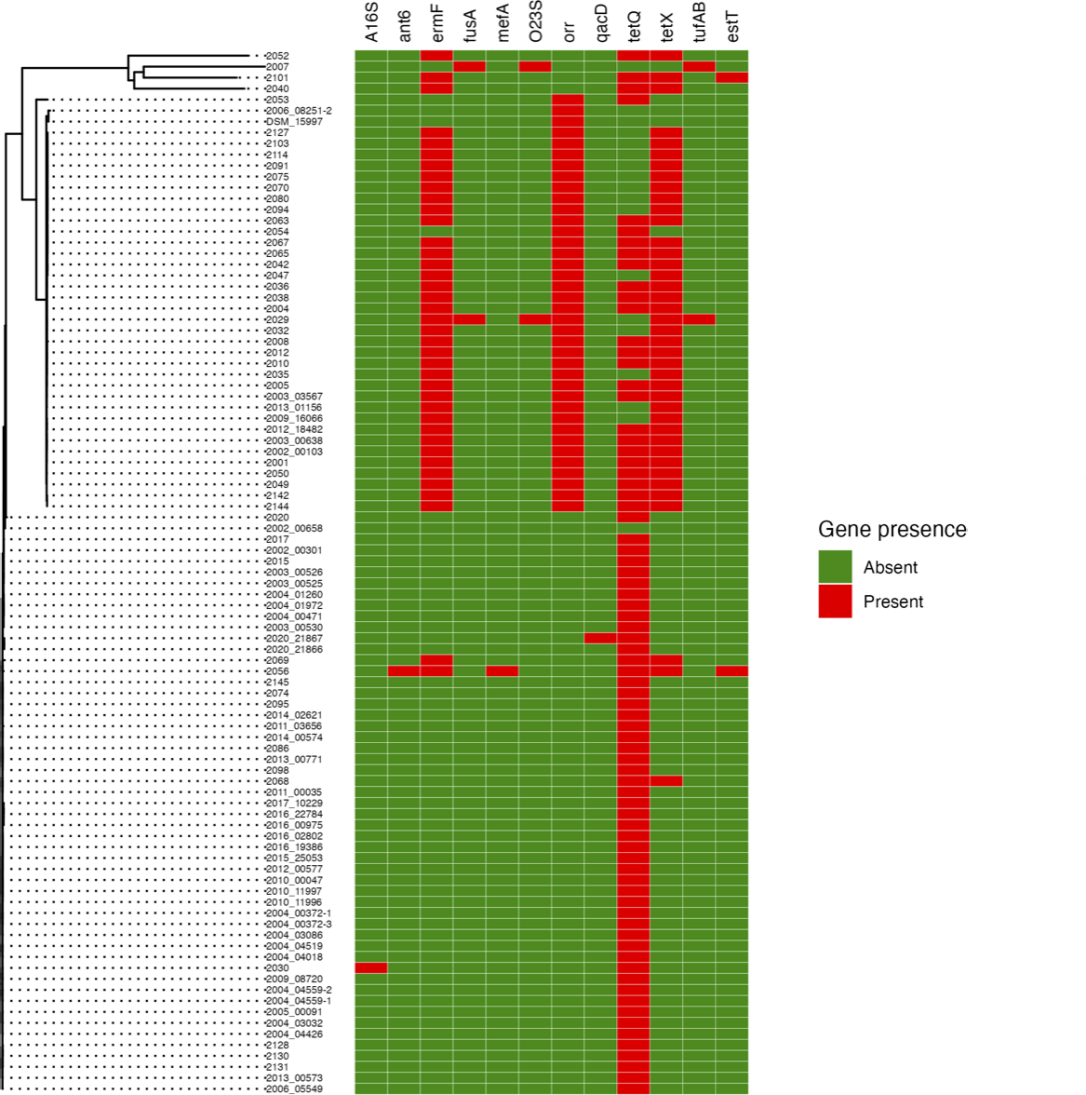
Distribution of resistance genes across ORT isolates. Phylogenetic tree generated with Parsnp 1.2 with a heatmap showing the presence (red) or absence (green) of 11 identified resistance genes across 94 ORT isolates. The heatmap highlights gene presence and absence patterns across isolates.

Through screening against the MEGARes, CARD, and AMRFinder databases, we identified a total of 12 resistance genes across all isolates (Fig. 6). Of these, four genes—*ermF* (macrolide resistance methyltransferase), *orr* (oxazolidinone resistance regulator), *tetQ,* and *tetX* (tetracycline resistance genes)—were the most common. Specifically, *ermF*, *orr*, and *tetX* were primarily associated with Clade O2, while *tetQ* was more ubiquitously present across the isolates. The remaining eight genes—A16S (mutant 16S rRNA methyltransferase), *ant6* (aminoglycoside nucleotidyltransferase), *fusA* (elongation factor G, fusidic acid resistance), *mefA* (macrolide efflux pump), O23S (mutant 23S rRNA methyltransferase), *qacD* (multidrug efflux pump), *tufAB* (elongation factor Tu, fusidic acid resistance), and *estT* (esterase, macrolide resistance)—were rare, each found in only one or two isolates. To further investigate the relationship between these resistance genes and phenotypic resistance profiles, we conducted a phi coefficient-based association analysis. This analysis helped identify several candidate genes with high correlations (phi > 0.7) to resistance against specific antibiotics. For example, *ermF* showed correlations with resistance to cefazolin (phi = 0.76), cefotaxime (phi = 0.74), ceftazidime (phi = 0.72), and tylosin (phi = 0.74). Similarly, *orr* was correlated with resistance to beta-lactam antibiotics such as cefazolin, cefotaxime, and ceftazidime, with phi coefficients above 0.7. *tetX* also showed correlations with cefazolin (phi = 0.77), cefotaxime (phi = 0.72), and tylosin (phi = 0.76).

#### Genomic context and co-localization of *tetX* and *ermF*

**Fig 7:**
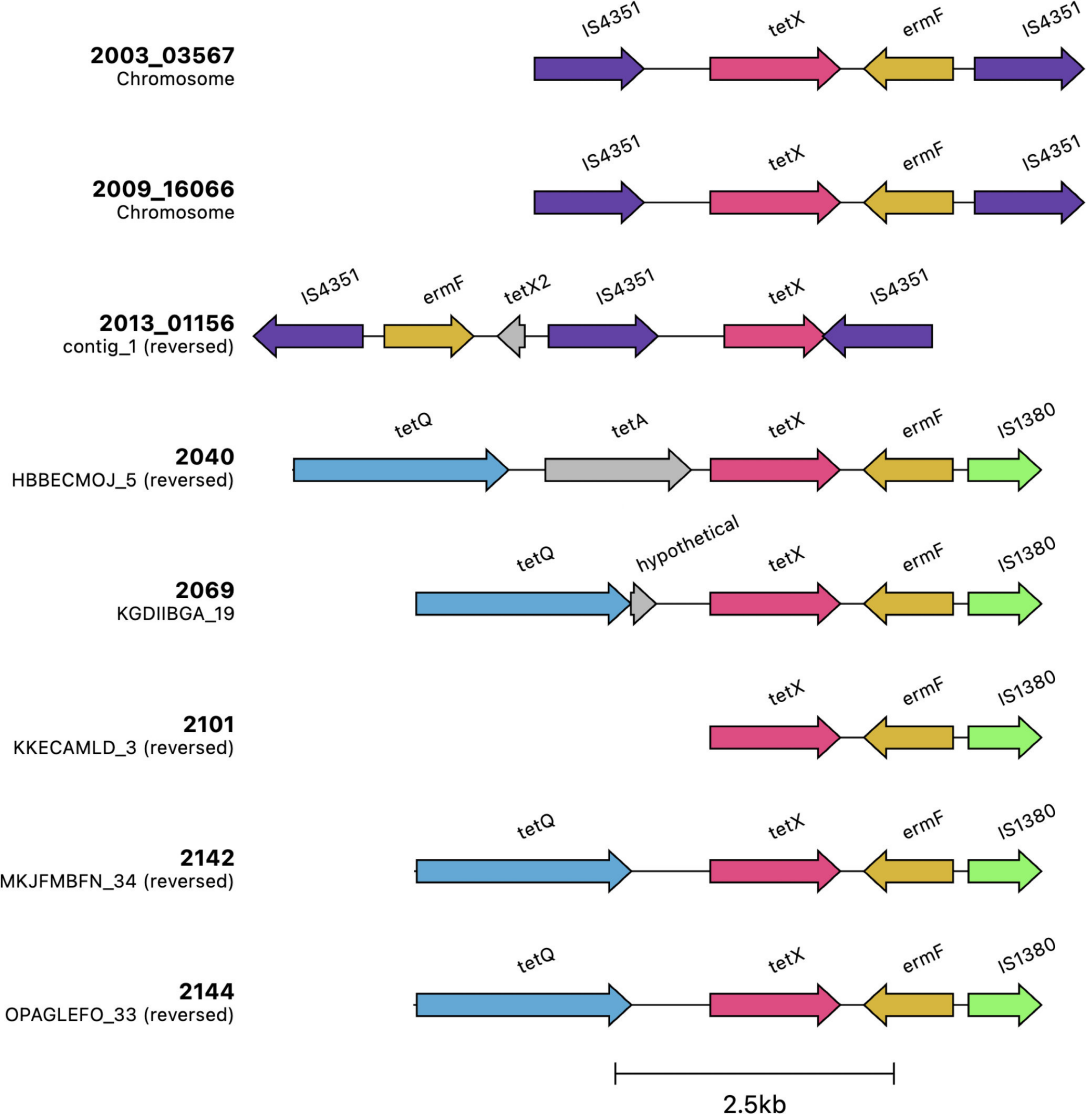
Genomic arrangements of *tetX* and *ermF/ermD* regions in ORT isolates. Each row is labeled with the isolate name (in bold) and a subtitle indicating the contig on which the arrangement is located, along with its orientation (reversed or not). This figure was generated using clinker.

Interestingly, the resistance profiles of *tetX* and *ermF* were nearly identical (Fig. 6), indicating potential co-selection due to their integration within a mobile genetic element. To investigate this, we analyzed their genomic locations across the isolates. In all 39 isolates where these genes were present (33 Austrian and 6 Hungarian), *tetX* and *ermF* were found adjacent to each other (Fig. S2). Moreover, in 33 of these isolates, the two genes were located on a short contig of 3 kb–5 kb, while in the remaining six isolates, they were found on a longer contig of apparent chromosomal origin. To determine whether this short contig is chromosomal or extra-chromosomal, we performed long-read sequencing on three isolates (2003_03567, 2009_16066, and 2013_01156) using Nanopore MinION technology. The assemblies revealed that isolates 2003_03567 and 2009_16066 were each resolved into a single-contig genome of approximately 2.5 Mb, confirming the chromosomal localization of *tetX* and *ermF*. In contrast, isolate 2013_01156 was assembled into eight contigs with a total genome size of approximately 2.77 Mb (Table S2). To further confirm the genomic context of these resistance genes, we aligned the assembled genomes of 2003_03567 and 2013_01156 against the complete chromosome of 2009_16066. The alignments demonstrated that all contigs from the multi-contig assemblies aligned perfectly to the 2009_16066 chromosome, further supporting that *tetX* and *ermF* are chromosomally encoded across these strains (Fig. S3). Further analysis revealed that in the three genomes we reassembled using Nanopore sequencing, *tetX* and *ermF* were consistently flanked on both sides by an IS30 family transposase (IS*4351*), indicating their presence within the Tn*4351* transposon. Notably, in isolate 2009_16066, IS*4351* was identified 22 times in the genome, with no sequence variation between copies. This repetitive nature of IS*4351* provides an explanation for the occurrence of the short contig containing *tetX* and *ermF* in isolates with lower sequencing coverage. In these cases, the repetitive flanking regions are difficult to assemble, leading to the formation of the short contig. Therefore, the peculiar occurrence of the short contig in some isolates can be attributed to the repetitive nature of IS*4351*. In addition, the six isolates from the Illumina-only assemblies where *tetX* and *ermF* were found on a longer contig showed both genes flanked by a single IS*1380* element (Fig. S2). These six isolates, together with those sequenced using Nanopore, encompass all the observed arrangements of the *tetX–ermF* region in our data set. To provide an overview of these configurations, we summarized them in Fig. 7. A BLAST search of the IS*4351*–*tetX*–*ermF*–IS*4351* sequence from isolate 2003_03567 against the nt database (≥90% query coverage, ≥90% identity) returned 14 matches (Table S3), including three from the avian respiratory pathogen *Riemerella anatipestifer* (two chromosomal, one plasmid) alongside hits in *Bacteroides fragilis*, *Segatella copri*, and several uncultured gut contigs. An analogous search with the *tetX–ermF*–IS*1380* sequence from isolate 2101 yielded 10 matches (Table S4), among them the avian respiratory pathogen *Riemerella columbina* and a previously sequenced *Ornithobacterium rhinotracheale* strain (FARPER-174b), in addition to multiple human gut Bacteroidetes.

#### Identification of resistance genes by GWAS

**Fig 8:**
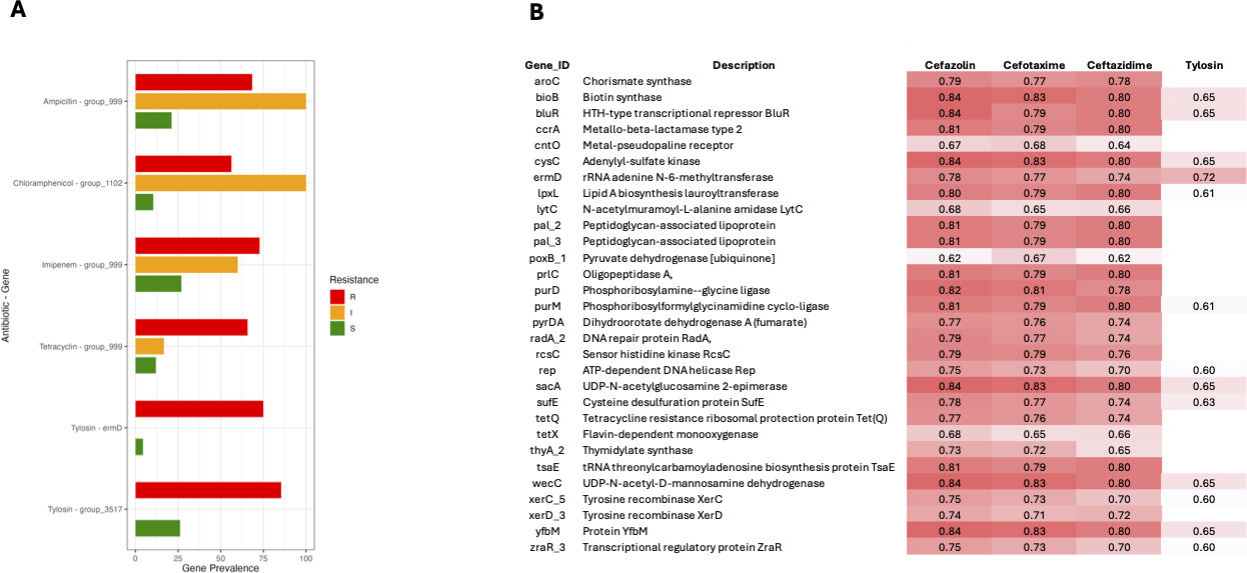
GWAS of resistance genes in ORT isolates. (**A**) Gene prevalence for significant associations identified using treeWAS, stratified by resistance (red), intermediate (yellow), and sensitive (green) phenotypes for specific antibiotics. (**B**) Heatmap of phi coefficients for gene-antibiotic resistance associations, with higher coefficients (darker red) indicating stronger correlations between gene presence and resistance phenotype.

For the GWAS, we used a combination of treeWAS and a phi coefficient-based association approach to identify genes linked to antibiotic resistance. We first employed treeWAS to analyze Panaroo’s gene presence/absence data. After filtering the significant genes for prevalence (>50% in resistant and <50% in sensitive isolates), we found six associations involving four genes, all of which showed consistently higher prevalence in resistant isolates compared to sensitive isolates (Fig. 8A). These genes were *group_999*, *group_1102*, *ermD*, and *group_3517*. The names beginning with “group” correspond to clusters assigned by Panaroo, which typically labels genes without a clear annotation as hypothetical proteins. However, *group_999* could be functionally annotated as encoding citrulline utilization hydrolase (CtlX), a protein primarily involved in citrulline metabolism. Upon reviewing the available literature, there is no evidence supporting its role in antibiotic resistance, suggesting that this association may be a false positive. Additionally, the treeWAS analysis identified *ermD* as being significantly associated with tylosin resistance. Although annotated as *ermD* by both Panaroo and Prokka, this gene matched *ermF* during our previous resistance gene screening performed with the MEGARes, CARD, and AMRFinder databases, so we will refer to it as *ermF/ermD* for consistency. To find other candidate resistance genes, we conducted a phi coefficient analysis to compute correlations between resistance profiles (R/I = 1, S = 0) and gene presence/absence data. We selected candidates with phi > 0.7, resulting in the identification of 30 putative gene-antibiotic resistance associations (Fig. 8B). This analysis is more prone to false positives due to the previously observed linkage between resistance profiles and phylogenetic clustering (Fig. 4B). Notably, one association that stands out is the metallo-beta-lactamase type 2 gene *ccrA*, linked to resistance against cefazolin, cefotaxime, and ceftazidime—all of which are beta-lactam antibiotics. This gene is the same as *orr*, previously identified through database screening (Fig. 6), so we will refer to it as *ccrA/orr* for consistency. In this phi coefficient analysis, we found again *ermF*/*ermD*, which is known to be involved in resistance to tylosin (40). Other important genes identified include tetracycline resistance genes *tetQ* and *tetX*, though neither was linked to tetracycline resistance in our data set. For all other candidate genes from Fig. 8B, we conducted literature searches for potential associations with resistance in other species but found no supporting evidence. Finally, to find potential SNPs associated with antibiotic resistance, we performed a treeWAS analysis using SNP data derived from the 94 isolates. This analysis identified 12 significant SNPs (Table 1). However, only two of these SNPs were non-synonymous, indicating a potential effect on protein function. These two non-synonymous SNPs constituted a single multiallelic variant (denoted by _1, _2 in the SNP name), located in the *gyrA* gene, which encodes the DNA gyrase subunit A. The mutation results in a Ser82Phe substitution, located within the canonical quinolone resistance-determining region (QRDR) and adjacent to Ser83—a well-known hotspot for resistance mutations in related species such as *Riemerella anatipestifer* (41). Although *gyrA* mutations in the QRDR are typically associated with fluoroquinolone resistance, including to nalidixic acid, this SNP was identified in our GWAS as being associated with ampicillin resistance. Correlation analysis showed low phi coefficients with nalidixic acid resistance (phi = 0.04 and 0.09 for the two alleles), indicating no notable association with this fluoroquinolone in our data set.

**TABLE 1:** Candidate SNPs identified through SNP-based GWAS using treeWAS GWAS in ORT isolates^*a*^

Antibiotic	SNP_ID	Ref_allele	Alt_allele	Gene_ID	STRING_Gene_ID	SNP_annotation
Ampicillin	snp_1076096	A	T			Intergenic
Ampicillin	snp_1947417_1	G	A	gyrA	Ornrh_1922	Non-synonymous, missense variant (Ser82Phe)
Ampicillin	snp_1947417_2	G	T	gyrA	Ornrh_1922	Non-synonymous, missense variant (Ser82Tyr)
Imipenem	snp_1893456	C	T	ORNRH_RS09035	Ornrh_1879	Synonymous, variant (Phe117Phe)
Imipenem	snp_1894444	G	A			Intergenic
Imipenem	snp_1894452_1	A	G			Intergenic
Imipenem	snp_314035	A	G	ORNRH_RS01460	Ornrh_0292	Synonymous, variant (Phe117Phe)
Tetracycline	snp_1453298	A	G	pepT	Ornrh_1419	Synonymous, variant (Gly195Gly)
Tylosin	snp_563723	T	G			Intergenic
Tylosin	snp_563790_1	A	G	ppk2	Ornrh_0546	Synonymous, variant (Gly256Gly)
Tylosin	snp_563793	T	A	ppk2	Ornrh_0546	Synonymous, variant (Pro255Pro)
Tylosin	snp_563868	T	A	ppk2	Ornrh_0546	Synonymous, variant (Pro230Pro)

^*a*^ Putative SNPs identified as candidates for antibiotic resistance in ORT isolates. Columns include the antibiotic for which the SNP was associated, SNP_ID, reference (Ref_allele) and alternate (Alt_allele) nucleotides, Gene_ID corresponding to the annotated gene, STRING_Gene_ID for cross-referencing with STRING database annotations, and SNP_annotation specifying whether the mutation is intergenic, synonymous, or non-synonymous. Full lists of isolate IDs carrying the alternate allele for each SNP are provided in Table S5.

#### Investigating the lack of correlation between *tet* genes and tetracycline resistance

To further investigate the lack of correlation for *tetQ* and *tetX* with tetracycline resistance, we checked whether any SNP could correlate with the resistance using the phi coefficient, but no associated SNPs (phi > 0.7) were found. To assess the potential presence of additional tetracycline resistance mechanisms, we analyzed the distribution of *tetQ* and *tetX* in our isolates. All 38 resistant and 6 intermediate isolates harbored at least one of these genes, while 37 of 50 sensitive isolates also carried one. We hypothesized that regulatory SNPs or other genomic variations might explain the observed lack of correlation between *tetQ* and *tetX* presence and tetracycline resistance. To test this idea, we examined subsets of isolates containing *tetQ*, *tetX*, or at least one of these two genes to identify any SNPs that might correlate with resistance. This focused analysis aimed to uncover potential regulatory changes or other genomic variations that could explain the observed resistance in isolates with these genes. Despite testing these subsets separately, no SNPs with a phi coefficient above 0.7 were identified in any of the three scenarios.

## DISCUSSION

In this study, we conducted the first large-scale genomic analysis of ORT isolates from Europe, specifically Austria and Hungary, to assess genetic diversity, clade distribution, and antibiotic resistance profiles. Our analysis identified two dominant clades, O1 and O2, encompassing most isolates from both countries. The absence of geographic clustering between Austria and Hungary suggests that these two clades circulate across both regions. The low genetic diversity within clades O1 and O2, with mean ANI values of 99.9% for O1 and 100% for O2, coupled with the stability of these lineages over three decades (1991–2023), supports a model in which ORT primarily spreads through clonal propagation rather than frequent recombination or repeated introductions of genetically distinct strains. This is consistent with previous multilocus sequence typing (MLST)-based analyses (42), which showed that ORT populations worldwide exhibit a highly clonal structure with limited genetic heterogeneity. While cross-border poultry trade and farm connectivity may facilitate transmission, the observed genetic stability over time suggests that these strains are highly fit and capable of maintaining dominance in poultry populations.

To date, the other extensive genomic study of ORT has been conducted in the USA by Smith et al. (17), who analyzed 157 clinical isolates from commercial turkey farms. The study by Smith et al. (17) identified four distinct phylogenetic clades, whereas our analysis identified two dominant clades (O1 and O2) in Austria and Hungary. Through a combined comparative genomic analysis, we established a clearer relationship between the European and US clades. Our findings show that Clade O2 is embedded within one of the US clades, suggesting a shared evolutionary origin or possible transcontinental transmission. In contrast, Clade O1 is distinct from all US isolates, representing a lineage specific to Europe. This clade exhibits a higher prevalence of resistance to six antibiotics—cefazolin, cefotaxime, ceftazidime, enrofloxacin, tetracycline, and tylosin—compared to Clade O2. One plausible hypothesis is that historical differences in antibiotic use between European and US poultry production created distinct selective pressures in the two regions. Testing this hypothesis will require (i) direct phenotypic susceptibility data for the US isolates and (ii) quantitative, farm-level records of antimicrobial use from both regions. Notably, Hungarian isolate 2053 does not cluster within Clades O1 or O2 but instead groups with the smallest US clade. This suggests that it belongs to a rare lineage that may be present at low frequencies in both regions. Isolate 2053 is also the only strain in our data set that harbors the *oatA* gene, which has been implicated in beta-lactam resistance (39). This observation is consistent with the findings of Smith et al. (17), who reported that *oatA* was uniquely associated with the same small US clade. Finally, both our study and that of Smith et al. (17) identified highly conserved genes involved in virulence and antibiotic resistance across ORT isolates. Genes associated with motility (*gldH*, *motB*), biofilm formation (*tabA*), and multidrug resistance (*macA*, *macB*, *mdtA*, *mdtC*, *norM*, *norR*, *yheI*, *yheS*) were prevalent in all isolates, underscoring their importance in ORT pathogenesis and persistence. These findings highlight key genetic factors that may contribute to ORT’s ability to colonize hosts, evade immune responses, and resist antimicrobial treatment across different geographic regions.

Our GWAS and database screening identified numerous potential candidate genes linked to antibiotic resistance. However, upon examining their known functions, it became apparent that many of these associations were likely false positives. To ensure the validity of our findings, we cross-referenced all candidate genes with existing literature on resistance mechanisms in other bacterial species. This approach allowed us to prioritize genes with well-established roles in antimicrobial resistance. Among the most reliable associations, we identified *ccrA/orr* as being linked to resistance against beta-lactam antibiotics such as cefazolin, cefotaxime, and ceftazidime (43). Similarly, *ermF/ermD* was associated with tylosin resistance, a finding that aligns with previous studies on macrolide resistance (40). Surprisingly, the presence of well-known tetracycline resistance genes such as *tetX* and *tetQ* did not correlate with tetracycline resistance in our data set. All resistant and intermediate isolates harbored at least one of these genes, suggesting that their presence is necessary for resistance. However, their variability in tetracycline-sensitive isolates—some of which carried *tetX* or *tetQ* while others lacked both—indicates that the mere presence of these genes is not sufficient to confer resistance. This lack of correlation between tetracycline resistance phenotype and genotype has also been observed in other bacterial species affecting poultry, such as *Enterococcus* spp. (44) and *Gallibacterium anatis* (45). However, while defective expression has been suggested as an explanation in other bacteria infecting humans (46) and pigs (47), it remains unknown whether the same regulatory mechanisms occur in ORT. Further transcriptomic and functional studies are necessary to determine if regulatory silencing, gene disruption, or other post-transcriptional modifications contribute to this pattern in ORT. Interestingly, *tetX* also showed correlations with resistance to cefazolin, cefotaxime, and tylosin, despite its known role in tetracycline resistance. However, these associations are likely an artifact of phylogenetic clustering rather than true functional links, underscoring the challenge of distinguishing genuine resistance markers from lineage effects in this data set. Similarly, the *gyrA* SNP identified in our analysis was correlated with ampicillin resistance, despite *gyrA* mutations typically being linked to fluoroquinolone resistance (48). This suggests that the role of this SNP in ampicillin resistance requires further investigation and could also be a false positive.

Interestingly, we observed that the *tetX* and *ermF*/*ermD* genes were often flanked by the insertion sequences IS*4351* and IS*1380* in isolates from Clade O1. IS*4351* is part of the composite transposon Tn*4351*, originally identified in *Bacteroides fragilis* (49), where it carries both *tetX*, which confers tetracycline resistance under aerobic conditions, and *ermF*, which mediates erythromycin resistance. This transposon has been described as a mobile genetic element capable of integrating into the chromosome, facilitating the spread of these resistance determinants within *Bacteroides*. Beyond *Bacteroides*, similar arrangements of *tetX* and *ermF*/*ermD* have been observed in other bacterial species. A recent large-scale genome analysis of human microbiota (50) revealed that *Butyricimonas phoceensis* harbors a transposase (IS*4351*) followed by *tetX* and *ermF*/*ermD*. However, no evidence from the literature indicates the presence of *tetX* and *ermF*/*ermD* flanked by either IS*4351* or IS*1380* in avian-associated bacteria. In our own BLAST searches, we found only 13 matches for the IS*4351*–tetX–ermF arrangement and 11 matches for the IS*1380*–tetX–ermF arrangement across public databases, highlighting the rarity of these configurations. Nevertheless, some of these matches included *Riemerella* spp., an avian respiratory pathogen, in which both IS elements were found associated with *tetX* and *ermF*/*ermD*. Although such arrangements are rare in avian bacteria, their presence in *Riemerella* suggests potential interspecies transfer, possibly from another respiratory pathogen. While the exact origin remains unclear, their presence in Clade O1 isolates points to an ancestral acquisition followed by vertical transmission within this lineage.

Despite identifying multiple potential associations through our various GWAS analyses, only the correlation of *ccrA/orr* with beta-lactam antibiotics and *ermF/ermD* with tylosin appeared to be the most robust. Some of these associations involve synonymous variants; while such changes can modulate expression via codon preference and tRNA availability (51), demonstrating a genuine regulatory role will require testing whether the observed codon-usage shift exceeds genome-wide expectations and corroborating any effects with transcriptomic data. More broadly, our ability to identify resistance genes in ORT was limited by the overall low genetic variability in our data set. To overcome this limitation, an integrated approach that combines genomics, transcriptomics, and proteomics is essential. By focusing on genes that are both differentially expressed and harbor mutations in resistant versus sensitive isolates, it will be possible to uncover novel resistance-associated loci.

Our findings have practical implications for ORT management, particularly in vaccine development and targeted antibiotic interventions. The identification of only two dominant strains across Austria and Hungary suggests that vaccines tailored to these specific clades may be broadly effective within the region. Additionally, the distinct resistance profiles observed between the two strains provide valuable information for refining treatment strategies. Current first-line therapy for ORT respiratory outbreaks in Europe still relies on broad-spectrum beta-lactams and tetracyclines. Field data and surveillance studies from Austria, Hungary, and Belgium report good *in vitro* activity of ampicillin-class drugs, chlortetracycline, and spectinomycin, but widespread resistance to gentamicin, nalidixic acid, and, notably, colistin (5, 8, 11, 52). Experimental studies in turkeys further suggest that enrofloxacin or florfenicol can outperform amoxicillin, while macrolides such as tilmicosin remain effective when administered early (53). Although colistin is not used for respiratory infections in poultry, its inclusion in resistance monitoring remains relevant due to the horizontal mobility of polymyxin resistance genes (54). These data underscore the importance of susceptibility-guided antibiotic use and align with our observation that Clade O1 and Clade O2 differ in beta-lactam and tetracycline resistance patterns. The restriction of Clade O1 to Europe, coupled with its unique resistance profile, may reflect incipient regional diversification. Overall, this study enhances the foundational understanding of ORT’s genomic diversity, clade structure, and resistance mechanisms in Europe, laying the groundwork for future surveillance, therapeutic, and vaccine strategies to control this important avian pathogen.

## Data Availability

The genome assemblies were submitted to NCBI under BioProject accession PRJNA1263164.

## SUPPLEMENTAL MATERIAL

Figure S1 aem.00569-25-s0001.pdf Phylogenetic tree of 94 *Ornithobacterium rhinotracheale* isolates and the reference strain DSM_15997, constructed using Parsnp and colored by collection year.

Figure S2 aem.00569-25-s0002.pdf Co-localization of *tetX* and *ermF/ermD* across ORT isolates.

Figure S3 aem.00569-25-s0003.pdf Whole-genome alignment of three ORT isolates sequenced using Nanopore MinION technology.

Table S1 aem.00569-25-s0004.xlsx Metadata and antibiotic resistance profiles for the 94 ORT isolates from Austria and Hungary and the reference genome DSM_15997.

Table S2 aem.00569-25-s0005.xlsx Genome assembly statistics for three ORT isolates sequenced using Nanopore MinION technology to investigate the genomic context of *tetX* and *ermF*/*ermD*.

Table S3 aem.00569-25-s0006.xlsx BLAST search results for the genomic region containing tetX and ermF/ermD flanked on both sides by IS4351 (from isolate 2003_03567, shown in Fig. 7), queried against the nt database.

Table S4 aem.00569-25-s0007.xlsx BLAST search results for the genomic region including tetX, ermF, and the flanking IS1380 element (from isolate 2101, shown in Fig. 7), queried against the nt database.

Table S5 aem.00569-25-s0008.xlsx Isolate identifiers carrying the alternate allele for each SNP listed in Table 1.

ASM does not own the copyrights to Supplemental Material that may be linked to, or accessed through, an article. The authors have granted ASM a non-exclusive, world-wide license to publish the Supplemental Material files. Please contact the corresponding author directly for reuse.
