# Supplementary material for: Genomic analysis reveals two dominant strains of Ornithobacterium rhinotracheale in Austria and Hungary with distinct multidrug resistance profiles
Source: Appl Environ Microbiol. 2025 Jul 21;91(8):e00569-25. doi: 10.1128/aem.00569-25 (PMC12366310; doi:10.1128/aem.00569-25)
Supplement: Figure S1 — Phylogenetic tree of 94 Ornithobacterium rhinotracheale isolates and the reference strain DSM_15997, constructed using Parsnp and colored by collection year. [file aem.00569-25-s0001.pdf]

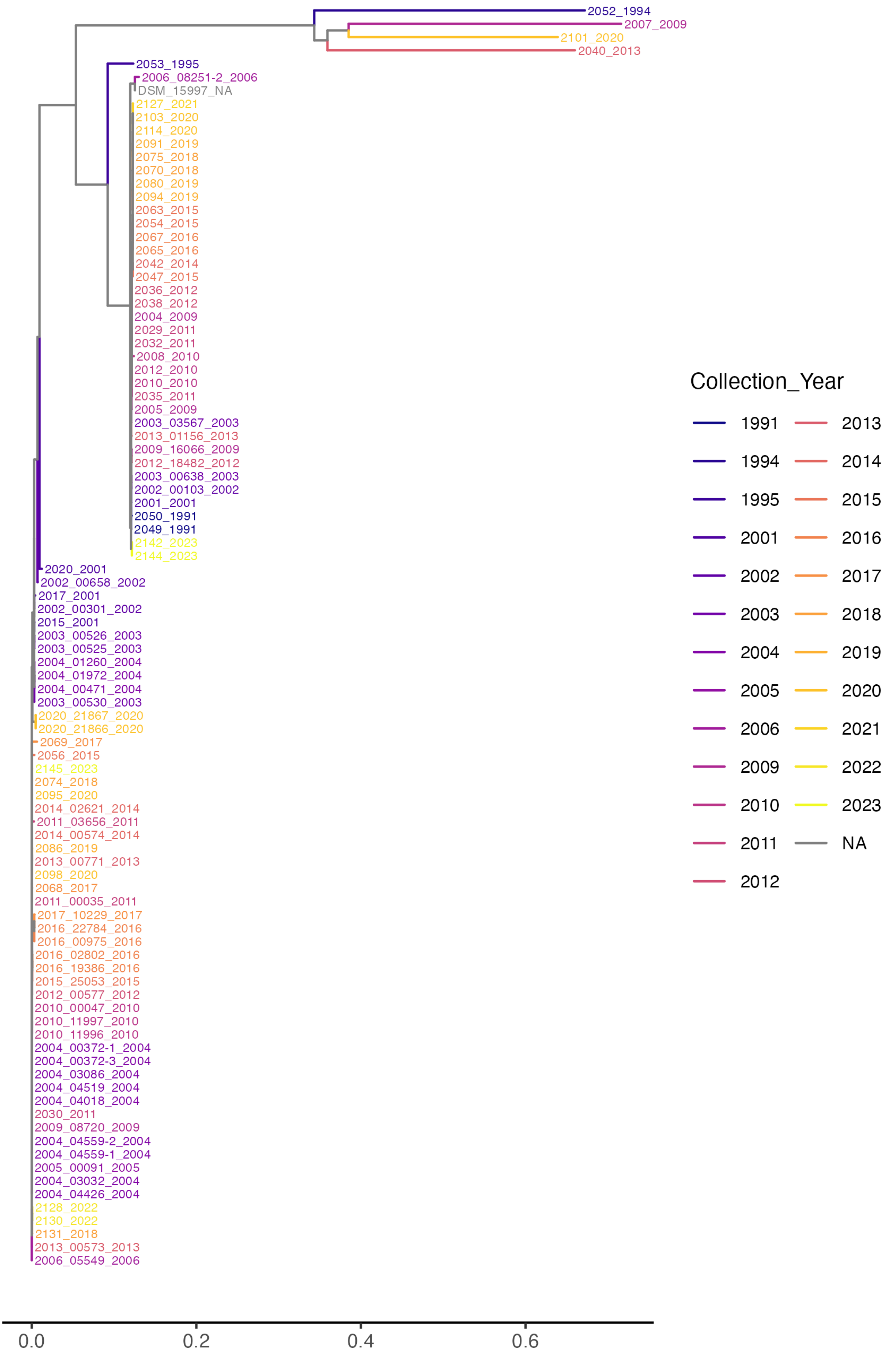


**Supplementary Figure 1.** Phylogenetic tree of 94 *Ornithobacterium rhinotracheale* isolates and the reference strain DSM_15997, constructed using Parsnp and colored by collection year.
