# Supplementary material for: Genomic analysis reveals two dominant strains of Ornithobacterium rhinotracheale in Austria and Hungary with distinct multidrug resistance profiles
Source: Appl Environ Microbiol. 2025 Jul 21;91(8):e00569-25. doi: 10.1128/aem.00569-25 (PMC12366310; doi:10.1128/aem.00569-25)
Supplement: Figure S2 — Co-localization of tetX and ermF/ermD across ORT isolates. [file aem.00569-25-s0002.pdf]

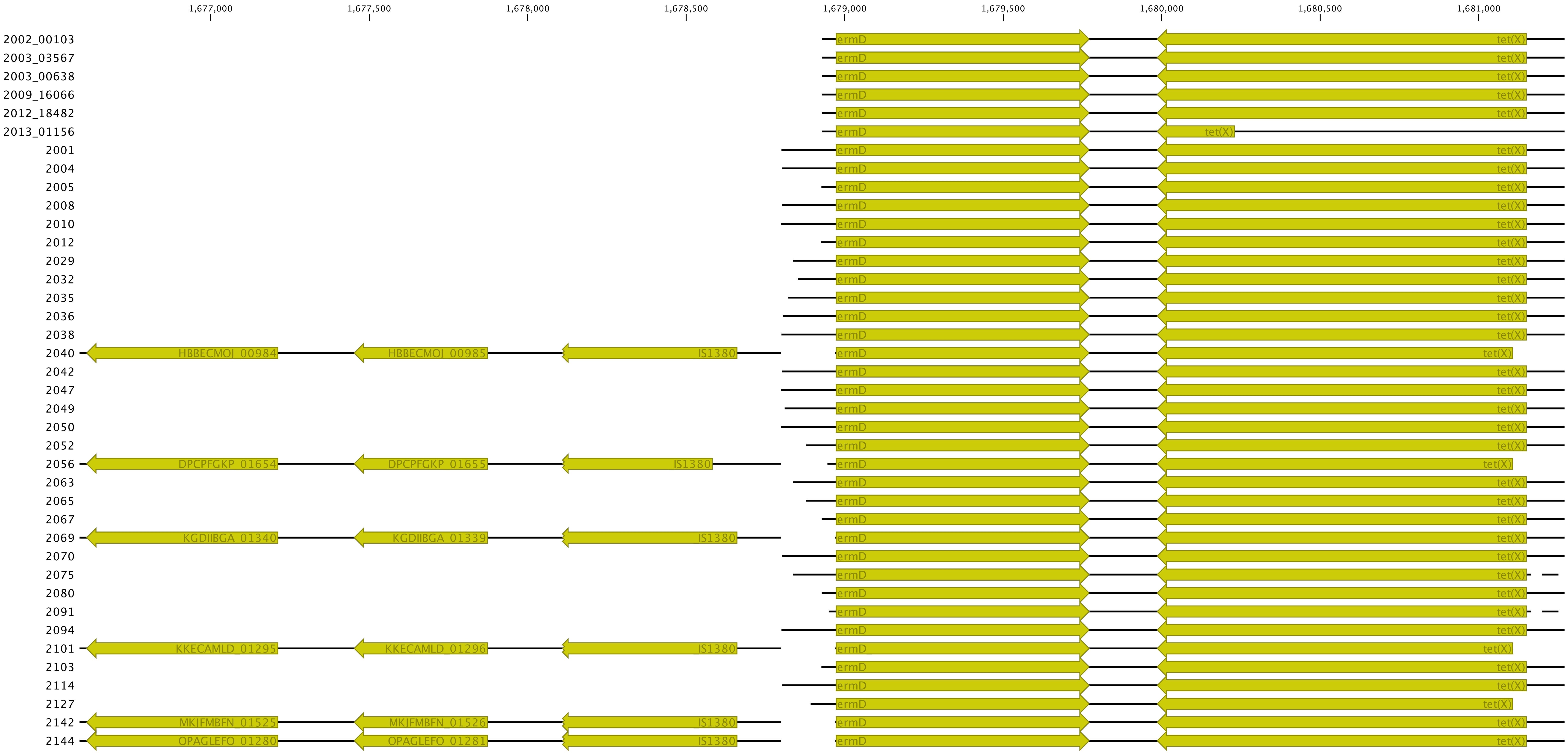

Supplementary Figure 2. Co-localization of *tetX* and *ermF*/*ermD* across ORT isolates.

Whole-genome alignment of all ORT isolates carrying both *tetX* and *ermF*, showing that the two genes are consistently adjacent to each other in every case. This supports their physical linkage across the dataset.
