# Supplementary material for: Genomic analysis reveals two dominant strains of Ornithobacterium rhinotracheale in Austria and Hungary with distinct multidrug resistance profiles
Source: Appl Environ Microbiol. 2025 Jul 21;91(8):e00569-25. doi: 10.1128/aem.00569-25 (PMC12366310; doi:10.1128/aem.00569-25)
Supplement: Figure S3 — Whole-genome alignment of three ORT isolates sequenced using Nanopore MinION technology. [file aem.00569-25-s0003.pdf]

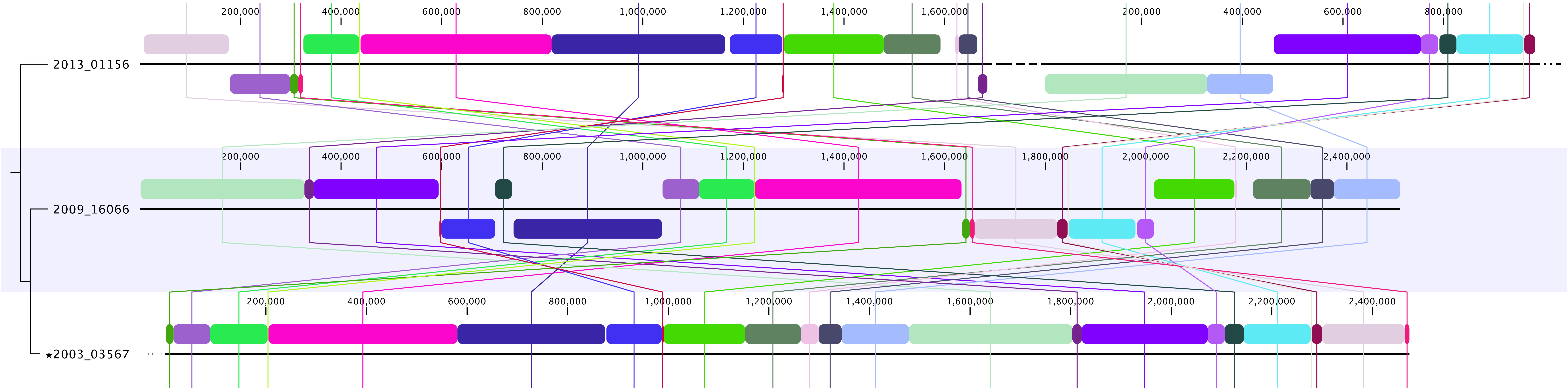

Supplementary Figure 3. Whole-genome alignment of three *ORT* isolates sequenced using Nanopore MinION technology.
Synteny blocks indicate regions of shared genomic organization among the isolates. The star (*) next to the isolate name **2003_03567** indicates that it was used as a reference in the alignment. The high degree of genome-wide collinearity suggests structural conservation across these isolates. The alignment was generated using the “Create Whole Genome Alignment” tool (CLC Genomics Workbench, Whole Genome Alignment plugin).
